# A new physiological medium uncovers biochemical and cellular alterations in Lesch-Nyhan disease fibroblasts

**DOI:** 10.1186/s10020-023-00774-8

**Published:** 2024-01-03

**Authors:** Paula Escudero-Ferruz, Neus Ontiveros, Claudia Cano-Estrada, Diane J. Sutcliffe, H. A. Jinnah, Rosa J. Torres, José M. López

**Affiliations:** 1 Institut de Neurociències, Universitat Autònoma de Barcelona, 08193 Cerdanyola del Vallès, Barcelona Spain; 2 Departament de Bioquímica i Biologia Molecular, Unitat de Bioquímica, Facultat de Medicina, Universitat Autònoma de Barcelona, 08193 Cerdanyola del Vallès, Barcelona Spain; 3 Department of Neurology, Emory University School of Medicine, 101 Woodruff Circle, 6305 Woodruff Memorial Building, Atlanta, GA 30322 USA; 4 Department of Human Genetics, Emory University School of Medicine, Atlanta, GA 30322 USA; 5 Department of Pediatrics, Emory University School Medicine, Atlanta, GA 30322 USA; 6 Center for Biomedical Network Research on Rare Diseases, Instituto de Salud Carlos III (ISCIII), 28029 Madrid, Spain; 7 Department of Biochemistry, La Paz University Hospital Health Research Institute, IdiPaz, 28046 Madrid, Spain

**Keywords:** Purine nucleotides, Folic acid, Lesch-Nyhan disease, ZMP, AICAR, Plasmax

## Abstract

**Background:**

Lesch-Nyhan disease (LND) is a severe neurological disorder caused by the genetic deficiency of hypoxanthine–guanine phosphoribosyltransferase (HGprt), an enzyme involved in the salvage synthesis of purines. To compensate this deficiency, there is an acceleration of the de novo purine biosynthetic pathway. Most studies have failed to find any consistent abnormalities of purine nucleotides in cultured cells obtained from the patients. Recently, it has been shown that 5-aminoimidazole-4-carboxamide riboside 5ʹ-monophosphate (ZMP), an intermediate of the de novo pathway, accumulates in LND fibroblasts maintained with RPMI containing physiological levels (25 nM) of folic acid (FA), which strongly differs from FA levels of regular cell culture media (2200 nM). However, RPMI and other standard media contain non-physiological levels of many nutrients, having a great impact in cell metabolism that does not precisely recapitulate the in vivo behavior of cells.

**Methods:**

We prepared a new culture medium containing physiological levels of all nutrients, including vitamins (Plasmax-PV), to study the potential alterations of LND fibroblasts that may have been masked by the usage of non-physiological media. We quantified ZMP accumulation under different culture conditions and evaluated the activity of two known ZMP-target proteins (AMPK and ADSL), the mRNA expression of the folate carrier SLC19A1, possible mitochondrial alterations and functional consequences in LND fibroblasts.

**Results:**

LND fibroblasts maintained with Plasmax-PV show metabolic adaptations such a higher glycolytic capacity, increased expression of the folate carrier SCL19A1, and functional alterations such a decreased mitochondrial potential and reduced cell migration compared to controls. These alterations can be reverted with high levels of folic acid, suggesting that folic acid supplements might be a potential treatment for LND.

**Conclusions:**

A complete physiological cell culture medium reveals new alterations in Lesch-Nyhan disease. This work emphasizes the importance of using physiological cell culture conditions when studying a metabolic disorder.

**Supplementary Information:**

The online version contains supplementary material available at 10.1186/s10020-023-00774-8.

## Introduction

**Fig. 1:**
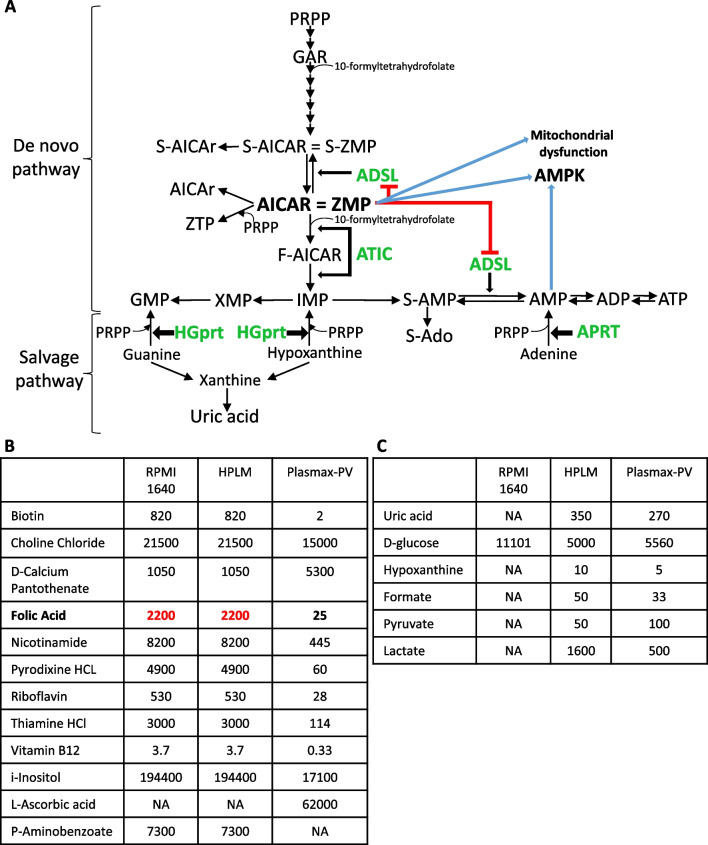
Purine metabolism in Lesch-Nyhan disease (LND) and cell culture media composition.** A** Scheme of the de novo and salvage pathways of purine nucleotide biosynthesis. The deficiency of HGprt enzyme in LND increases the levels of guanine, hypoxanthine, and PRPP and accelerates the de novo pathway producing high levels of uric acid. Adenine phosphoribosyltransferase (APRT) catalyses the synthesis of AMP from PRPP and adenine. There are two enzymes in the de novo pathway that incorporate 10-formyltetrahydrofolate: GAR transformylase (GART), and 5-aminoimidazole-4-carboxamide ribotide transformylase-IMP cyclohydrolase (ATIC). ZMP can inhibit the bifunctional enzyme adenylosuccinate lyase (ADSL), induce AMP-activated protein kinase (AMPK) activation and mitochondrial dysfunction. **B**, **C** Formulations of Roswell Park Memorial Institute (RPMI) medium 1640, Human Plasma-Like Medium (HPLM) and Plasmax with physiological vitamins (Plasmax-PV). Vitamin concentrations are expressed in nM (**B**), whereas the concentrations of other important components are in μM (**C**). *NA* not available

Lesch-Nyhan disease (LND) is a severe neurological disorder caused by mutations in the *HPRT1* gene, located on the X chromosome, that leads to the deficiency of hypoxanthine–guanine phosphoribosyltransferase (HGprt) enzyme. HGprt enzyme is involved in the salvage synthesis of purines and its deficiency causes guanine, hypoxanthine, and phosphoribosyl pyrophosphate (PRPP) accumulation in the cells (Fig. 1A). Since hypoxanthine and guanine cannot be recycled, they are degraded to uric acid leading to hyperuricemia (Fig. 1A) (Jinnah et al. 2006). Moreover, there is an acceleration of the de novo purine biosynthetic pathway thus increasing the production of uric acid (Fig. 1A) (Rosenbloom et al. 1968; Fu et al. 2015).

Besides hyperuricemia, LND patients present macrocytic anemia and several neurological dysfunctions including dystonia, spasticity, cognitive disability, self-injurious behavior and a marked reduction of dopamine content in the basal ganglia (Anderson and Ernst 1994; Visser et al. 2000; Jinnah et al. 2006). The administration of allopurinol from birth controls hyperuricemia but does not ameliorate the neurological problems.

In addition to the classic disease, residual levels of HGprt activity have been correlated with LND variants that show milder or absent neurological clinical features. LND variants are sub-divided in two different categories depending on the presence or absence of the neurological phenotype: HGprt-related neurological dysfunctions (HND) and, the mildest phenotype, HGprt-related hyperuricemia (HRH) (Fu et al. 2015). There are no significant differences in serum uric acid levels among LND, HND and HRH, indicating that uric acid is not responsible of the neurobehavioral problems in LND (Jinnah et al. 2010).

Recently, it has been shown that 5-aminoimidazole-4-carboxamide riboside 5ʹ-monophosphate (ZMP, also known as AICAR), an intermediate of the de novo purine biosynthesis pathway (Fig. 1A), accumulates in LND fibroblasts (López et al. 2020). Furthermore, Z-metabolites derivatives (AICAr and AICA) are found at high levels in the urine from LND patients and its variants (HND and HRH) with a higher ratio AICAr/AICA in patients presenting neurological alterations (LND and HND), and AICAr is present in the cerebrospinal fluid of LND patients but not in control individuals (López et al. 2020). Prior to that, it was reported that Z-nucleotides (ZMP and its triphosphate derivate ZTP) accumulate in erythrocytes from LND patients (Sidi and Mitchell 1985; Ceballos-Picot et al. 2015) and ZMP in the brain of *HPRT1* knockout mice (Tschirner et al. 2016).

Altogether, the previous studies suggest that ZMP accumulation may have a key role in the progression of LND disorder. Several known targets of ZMP have been proposed to participate in LND pathogenesis (López 2008): inhibition of mitochondrial oxidative phosphorylation (Guigas et al. 2007), inhibition of the bifunctional enzyme adenylosuccinate lyase (ADSL) (Sabina et al. 1982, 1985) and activation of the AMP-activated protein kinase (AMPK) (Corton et al. 1995) (Fig. 1A). To our knowledge these targets have not been studied in cells obtained from LND patients.

Importantly, ZMP accumulation in fibroblasts is only observed when the cell culture medium contains physiological levels of folic acid (FA: 25 nM) (López et al. 2020), which strongly differs from FA present in regular commercial media (2200 nM) (Fig. 1B). FA and its derivatives are relevant to study the pathogenesis of LND since two enzymes in the de novo purine biosynthetic pathway use 10-formyltetrahydrofolate as a substrate: GAR transformylase (GART), and 5-aminoimidazole-4-carboxamide ribonucleotide transformylase-IMP cyclohydrolase (ATIC), being ATIC more sensitive to folic acid depletion (Fig. 1A) (Baggott and Tamura 2015). To avoid nutrient exhaustion and to increase cell proliferation, most of the commercial media contain high non-physiological levels of nutrients such as glucose, glutamine, vitamins, and amino acids (Fig. 1B, C). While these ingredients promote cell growth, they do not precisely recapitulate the in vivo behavior of cells (Cantor 2019; Ackermann and Tardito 2019; Golikov et al. 2022a) and may have a great impact on studies of metabolic diseases. Recently, Human Plasma Like Media (HPLM) (Cantor et al. 2017) and Plasmax media (Vande Voorde et al. 2019) have been used to overtake commercial media flaws. Plasmax and HPLM contain a reduced amount of glucose and several amino acids compared to standard media to mimic plasma concentrations and are supplemented with a variety of components that are present in plasma but are omitted from RPMI and other commercial media such as lactate, uric acid, formate and hypoxanthine (Fig. 1C). Considerable differences in cancer cell metabolism have been reported when using Plasmax or HPLM instead of commercial media that may lead to more accurate and rigorous conclusions (Cantor et al. 2017; Vande Voorde et al. 2019; Hennequart et al. 2021; Moradi et al. 2021).

Unfortunately, HPLM and Plasmax contain very high levels of vitamins, including folic acid. We show here that HPLM and Plasmax containing high folic acid (2200 nM) do not induce ZMP accumulation in LND fibroblasts. In this article, we describe a new cell culture medium, Plasmax-PV, which is Plasmax medium containing physiological levels of vitamins, and we believe this medium represents an improvement to understand the pathophysiology of Lesch-Nyhan disease. We describe that cell growth is critical to detect ZMP accumulation in LND fibroblasts. Moreover, we reveal several alterations in LND fibroblasts that may have been masked by the usage of non-physiological media. Although there are no differences between control and LND fibroblasts in the activity of two well-known ZMP-target proteins (AMPK and ADSL) and in the activity of mTORC1, we observe that LND fibroblasts have an increased glycolytic capacity, a higher expression of the folate carrier *SLC19A1*, and some functional alterations, such a decreased mitochondrial potential and impaired migration capacity, compared to controls.

## Materials and methods

### Research subjects

Subjects with HGprt deficiency were diagnosed according to accepted criteria. Subjects with LND had < 2% HGprt enzyme activity, or a genetic variant in the *HPRT1* gene predicting null activity, along with evidence for overproduction of uric acid and a characteristic neurobehavioral phenotype (Jinnah et al. 2006). Milder forms of the disease, named Lesch-Nyhan variants, exhibited some residual HGprt activity, along with a less severe clinical phenotype (Jinnah et al. 2010). The variants were divided into two groups: those with HND had overproduction of uric acid along with some neurobehavioral problems, while those with HRH had overproduction of uric acid without obvious neurobehavioral problems.

### Culture of human fibroblasts

Primary skin fibroblasts from patients with LND and control individuals were collected as previously described (Fu et al. 2015) and maintained in RPMI without folic acid (FA) (Sigma-Aldrich, R1145) supplemented with 15% of FBS (Sigma-Aldrich, T7524), 2200 nM of filtered FA (Sigma-Aldrich, F8758), 1% of L-glutamine (Gibco, 250381), 1% of Pen/Strep (Gibco, 250381) and 0.2% of sodium bicarbonate (Sigma-Aldrich, S8761). The day before treatment, 120,000 cells were seeded in a 100 mm dish. The next day, dishes were washed with PBS and then maintained for 5 days with some of the following media: RPMI with 2200 nM of FA, RPMI with 25 nM of FA, RPMI with 25 nM of FA and 350 µM of uric acid or HPLM (Gibco, A4899101). The media were changed every two or three days. For Plasmax-PV experiments, cells were washed with PBS and maintained for 7 days in RPMI or Plasmax-PV medium with 2200 or 25 nM of FA and the specified FBS percentage. In these experiments media was changed daily after the third day of seeding the cells.

Plasmax, without any vitamin was prepared as described by its manufacture (Cell Culture Technologies, Gravesano, Switzerland) and sodium hydrogen carbonate added to a final concentration of 26.5 mM (Merck, 1.06329). A stock of vitamins 100 × containing biotin (Sigma-Aldrich, B4501), choline chloride (Sigma-Aldrich, C7527), inositol (Sigma-Aldrich, I7508), d-calcium pantothenate (Sigma-Aldrich, 21210), pyridoxal (Sigma-Aldrich, 271748), riboflavin (Sigma-Aldrich, R4500), thiamine HCl (Sigma-Aldrich, T4625), vitamin B12 (Sigma-Aldrich, V2876) was prepared and kept at -20 °C. The specific final concentration of each vitamin is presented in Fig. 1B. Plasmax-PV was prepared by supplementing Plasmax with the appropriate amount of FBS (15% or 2.5% final concentration), l-glutamine (650 µM), 1% of Pen/Strep, the stock of vitamins 100× to a final 1× concentration, ascorbic acid (62 µM) (Sigma-Aldrich, A92902) and 2200 nM or 25 nM of filtered FA (Sigma-Aldrich, F8758). Plasmax-PV was stored at 4 °C and protected from the light.

For AICAr treatment experiments, cells were maintained for 7 days with Plasmax-PV medium containing 25 nM of FA and AICAr (Toronto Research Chemicals, TRC, A611700) was added at the appropriate concentration during the last 24 h.

### Cell growth

Fibroblasts were counted at the day of seeding and at the day of collecting the cells, after trypan blue staining, with a Neubauer chamber. The doubling time was estimated applying the following formula, which considers a linear regression during the exponential growth phase:

$${\text{Doubling}}\;{\text{time }} = \frac{{{\text{Ln }}2{ } \times {\text{ Time}}\left( {{\text{days}}} \right)}}{{{\text{Ln }}\frac{{{\text{Final}}\;{\text{cell}}\;{\text{concentration}}}}{{{\text{Initial}}\;{\text{cell}}\;{\text{concentration}}}}{ }}}$$

### Urine samples from LND and control individuals

A morning urine sample was obtained from control individuals (N = 6), patients with LND (N = 14), HRN (N = 8) or HRH (N = 2) with the following average ages, in years, for each group: control (14.8 ± 8.0), LND (30.9 ± 14.7), HRN (17.5 ± 12.0) and HRH (24.5 ± 17.8). One urine sample from a patient with ADSL deficiency was kindly given by Rafael Artuch, Laboratorio de Bioquímica, Hospital Sant Joan de Déu, Barcelona, Spain. Urine samples were aliquot and kept at − 80 °C until the day of the experiment. The day of the assay urine samples were thawed on ice and 180 µl were centrifugated at 12,000×*g*, 5 min at 4 °C. Creatinine was measured in a multichannel autoanalyzer (Modular P800, Roche, Mannheim, Germany; and Hitachi 704, Hitachi, Tokyo, Japan) or with a creatinine colorimetric assay kit (Cayman Chemical, 500701) for further results normalization.

### Purine measurement

For purine determinations, fibroblasts were detached by trypsinization, counted in a Neubauer chamber, and resuspended in 0.4 N PCA. For urine samples, 150 µl of supernatant were used and 5 µl of perchloric acid (PCA) 60% (6 N) were added to obtain a final concentration of 0.2 N PCA. The samples were kept on ice for 15 min and then centrifuged at 12,000×*g* for 5 min at 4 °C. Pellet obtained from fibroblast extraction was stored at − 20 °C for later protein quantification and supernatant from fibroblasts or urines was neutralized with 5 M potassium carbonate (Sigma-Aldrich, 209619) and filtered through PVDF micro spin filters (Thermo Scientific, F2517-5) via centrifugation at 10,000×*g* for 10 min at 4 °C. The supernatants filtered were kept at − 80 °C for HPLC determination.

HPLC coupled to an UV detector was used for purine determinations. Analytes were separated using reverse-phase ion-pair chromatography on an Atlantis T3 column (Waters, 186003729). The optimized method for nucleotides separation and quantification consist on this sequence of stepped gradient of buffer A (10 mM of ammonium acetate (Sigma-Aldrich, A1542) and 2 mM of tetrabutylammonium phosphate monobasic solution (Sigma-Aldrich, 268100) pH5) and buffer B (10 mM of ammonium phosphate (Sigma-Aldrich, 09709), 2 mM of tetrabutylammonium phosphate, and 25% of acetonitrile (J.T. Baker, 76045) pH7) as follows: 100% of buffer A for 10 min, a linear gradient of buffer B up to 75% over 10 min, 9 min at 75% buffer B, a linear gradient to 100% buffer B over 3 min, 8 min at 100% buffer B, a linear gradient to 100% A in 1 min and finally 10 min maintained at 100% buffer A. The identification of purines was made by comparing their retention times to known standards and were quantified at 254 nm in a deuterium lamp. Sample analysis was performed by EZ Chrom Elite/ELITE UV–VIS software.

To determine intracellular concentration of nucleotides, skin fibroblasts single cell volume (2 × 10^–6^ μl) was calculated empirically.

### Protein quantification

Protein quantification was determined by using a commercial Micro BCA Protein Assay Kit (Pierce, 23235). Protein extracts were resuspended in 2% SDS and incubated overnight at 37 °C to completely dissolve the protein pellet. 1:100 dilutions were made and incubated with reagent solution at 37 °C for 2 h. After that, absorbance was measured at 625 nm in a PowerWave XS spectrophotometer (Bio-Tek). A standard curve with increasing concentrations of BSA was prepared and used for quantification.

### Western blotting

Total cell lysate was obtained by addition of 250 µl lysis buffer A (50 mM Tris, 2% SDS, pH 6.8) to one 100 mm dish of cultured cells and heating at 100 °C for 5 min. For the mitochondrial and cytosolic fraction 2 plates of human fibroblasts (3 × 10^6^ cells approximately) were scrapped in 100 µl of buffer B per plate (20 mM HEPES pH7.5, 250 mM sucrose, 0.1 mM EDTA, including protease inhibitors: 0.1 mg/ml leupeptin, 0.01 mg/ml aprotinin, 1 mM PMSF; and phosphatase inhibitors: 50 mM NaF, 5 mM sodium pyrophosphate, 10 mM βGP, 1 mM sodium orthovanadate). Then, the cells were lysed by passing through a 20-gauge syringe 15 times, centrifuged 800×*g* for 10 min at 4 °C and the supernatant was collected. The pellet was resuspended in 100 µl of buffer B and re-homogenize and spin as before. Both supernatants were taken and combined and centrifuged at 11,000×*g* during 15 min at 4 °C. The supernatant was taken and corresponded to the cytosolic fraction, and the pellet was resuspended in 500 µl of buffer B and centrifuged again at 11,000×*g* during 15 min at 4 °C. The pellet was resuspended in 60 µl of buffer B and corresponded to the mitochondrial fraction. Extracts were preserved at − 20 °C and protein quantification was determined as described before.

Samples collected (80 µg protein in total extracts and 30 µg in cytosolic and mitochondrial extracts) were denatured in Laemmli sample buffer at 100 °C for 5 min. Electrophoretic separation was performed in a 10% polyacrylamide gel and proteins were transferred to a PVDF membrane in transfer buffer (25 mM Tris, 192 mM glycine, 10% methanol) at 90 V for 90 min.

After transference, the membrane was washed with TBST (25 mM Tris, 135 mM NaCl, pH 7.5 and 0.1% Tween20) and blocked for 60 min with 5% dried skimmed milk in TBST, washed several times with TBST, and then incubated overnight at 4 °C with primary antibodies for AMPK (Cell Signaling, 2532; 1:1000), pAMPK (Cell Signaling, 2531; 1:1000), VDAC (Cell Signaling, 4866S; 1:1000), HPRT (Santa Cruz, 376938; 1:150), pRAPTOR (Cell Signaling, 2083; 1:1000), RHEB (Abnova, H00006009-M01; 1:500), pS6 (Cell Signaling, 4856; 1:1000), ATF4 (Cell Signaling, 11815S, 1:500), β-actin (Sigma-Aldrich, A1978; 1:500), diluted in TBST containing 5% BSA (Sigma-Aldrich, A9647). Antibody binding was detected next morning by washing the membrane with TBST several times and incubating for 1 h at room temperature with horseradish peroxidase-coupled (HRP) secondary antibody (anti-rabbit HRP from Invitrogen, 31460; anti-mouse HRP from BD Bioscience, 554002) diluted 1:3.000 in TBST containing 5% dried skimmed milk.

Membrane was washed several times with TBST and signal was detected by chemiluminescence in Odyssey Fc Imaging System, LI-COR. Resulting bands were analyzed and quantified using the program Image-J.

### Cell migration by scratch assay

The day before treatment, 160,000 cells were seeded in a 100 mm dish with RPMI media containing 2200 nM FA and 15% FBS. Before the treatment, dishes were washed with PBS and then maintained for 6 days in Plasmax-PV medium with 2200 or 25 nM of FA and 15% FBS. Medium was changed from the third day on in a daily manner. On the 6th day, cells were detached with trypsin and 650,000 of each condition were seeded in a 6-well plate with the appropriate Plasmax-PV medium. Cells were left at the 37 °C incubator for approximately 6 h allowing them to properly attach and create a confluent monolayer. Then, the cell monolayer was scraped in a straight line with a p10 pipet tip to create a “scratch”. Wells were washed with PBS to remove the debris and smooth the edge of the scratch. PBS was replaced by Plasmax-PV media containing either 2200 nM or 25 nM FA and 2.5% of FBS to avoid cell growth and to assess only cell migration. The plate was placed immediately under a Zeiss LSM980 microscope (Carl Zeiss Microscopy GmbH, Oberkochen, Germany). Six referent points per each well were marked thanks to the Zeiss software associated to the microscope. For each referent point one image was acquired every 30 min for 24 h. Images were processed with ZEN Black 3.0 SR FP1 software (Carl Zeiss Microscopy GmbH, Oberkochen, Germany) and analyzed by ImageJ. For each well, distances (µm) between one side of scratch and the other were measured from images taken at 0, 5 and 10 h post-scratch by setting up the scale and using the straight lines tool in Image-J. Six referent points per condition were taken and the average of ten measurements for each point was considered the distance heal at the specific time point and culture condition.

### Mitochondrial respiration and glycolytic capacity by seahorse analysis

Agilent seahorse XF cell MitoStress Test (Agilent, 103010-100) was used to assess mitochondrial function by determination of key parameters including basal respiration, maximal respiration and ATP production expressed as oxygen consumption rate (OCR). The extracellular acidification rate (ECAR) was also measured. ECAR is primarily a measure of lactate production and can be equated to the cellular glycolytic rate.

Skin fibroblasts were cultivated in 100 mm dishes with the specific Plasmax-PV or RPMI medium. On the 6th day of culturing cells, fibroblasts were detached and seeded in the Seahorse XFp cell culture miniplate (Agilent, 103022-100). Three replicates of 7000 cells/well were seeded in 80 µl of RPMI or Plasmax-PV media. This optimal cell density per cell line was determined by a titration assay. The two wells on the extremes of the plate were filled only with medium and used as background correction. Cells were left at room temperature for 60 min and then transferred to the 37 °C incubator for 18 h. The day prior to the assay, the XF sensor cartridge (Agilent, 103022-100) was hydrated with calibrant solution (Agilent, 103022-100) and incubated in a non-CO_2_ incubator at 37 °C overnight. The day of the assay, 45 min prior to the MitoStress Test, media in the cell plate was changed from Plasmax-PV or RPMI to Agilent Seahorse XF DMEM medium pH 7.4 (Agilent, 103575-100) lacking phenol red dye and bicarbonate and supplemented with 10 mM glucose (Gibco, A2494001), 1 mM pyruvate (Sigma-Aldrich, S8636) and 2 mM glutamine (Gibco, 250381). The cell plate was kept in a non-CO_2_ 37 °C incubator to get rid of the CO_2_ that could interfere with the pH measurement. It was previously assessed that 45 min of incubation with seahorse media that contains high levels of FA did not reverse ZMP accumulation in LND fibroblasts induced by Plasmax-PV and low FA. On the meantime, oligomycin (1.0 μM), FCCP (1.0 μM), and rotenone/antimycin A (0.5 μM) were added to the hydrated sensor cartridge. The sensor cartridges were loaded into the Seahorse Analyzer and calibration was performed for 15 min. After calibration, the sensor cartridges were replaced by the plates with cells and the measurement program was proceeded to obtain the following OCR parameters: basal mitochondrial respiration (value 3–value 12) and maximal respiration (maximal value within 7/8/9–value 12) and ATP production (value 3–value 6) and extracellular acidification rates (ECAR) as a measure of glycolysis. OCR and ECAR parameters were normalized by cell number per field of view, measured by a Hoestch staining in the same plate and later quantified by Image-J. 8 pictures per each well were acquired and the average number of cells was taken for normalization. OCR parameters were presented as (pmol/min)/cells in the field of view and ECAR was presented as fold change pre/post oligomycin. The raw data was processed by Seahorse Wave Desktop software (Agilent Technologies) and further analysed by Excel and Graphpad.

### Mitochondrial membrane potential (DilC staining)

This assay was performed following the instructions detailed in the MitoProbe DilC1(5) Assay kit for Flow Cytometry (Invitrogen, M34151). For each sample, 3 × 10^5^ cells were resuspended in 500 µL of PBS. As a control of mitochondrial depolarization samples were incubated for 5 min with 50 µM of CCCP, final concentration, at 37 °C. Then, DilC was added to achieve a final concentration of 10 nM and incubated for 30 min at 37 °C, 5% CO_2_. Cells were washed and resuspended in 250 µl of PBS. Each tube was analyzed by flow cytometry with 633 nm excitation and suitable filter for Alexa Fluor 633dye.

### qRT-PCR

Total RNA was extracted from control and LND fibroblasts cultivated with different media using the Maxwell RSC simply RNA Cells Kit (Promega, AS1390) following the manufacturer’s instructions. This procedure includes DNase I treatment to prevent DNA contamination. Reverse transcription (RT) was carried out with iScript cDNA Synthesis Kit (Biorad, 1708891) using 1 µg of total RNA. RT was performed in a thermal cycler as follows: 5 min at 25 °C for priming, 20 min at 46 °C for RT, and 1 min at 95 °C for RT inactivation.

Quantitative PCR reactions took place in a 384-well CFX384 Touch Real-Time PCR Detection System (Biorad), using 5 µl of iTaq Universal SYBR Green Supermix (Biorad) and 0.4 µM of forward and reverse primers in a final volume of 10 µl. Every reaction was made by triplicate as follows: pre-heating 3 min at 95 °C, followed by 40 cycles of 10 s at 95 °C, 30 s at 60 °C, and for the melt curve starting at 65 °C and increasing 0.5 °C every 5 s until reaching 95 °C.

Primers used were based on the ones reported by Lee et al. (Lee et al. 2021). *SLC19A1* was the studied gene and *RPS11* and *TPT1* were used as reference genes. Primer sequences are:

**Table:** 

*SLC19A1*	Forward: CCTCGTGTGCTACCTTTGCTT
	Reverse: TGATCTCGTTCGTGACCTGC
*RPS11*	Forward: CCGAGACTATCTGCACTACATCC
	Reverse: GTGCCGGCAGCCTTG
*TPT1*	Forward: CACCTGCAGGAAACAAGTTTC
	Reverse: GTCACACCATCCTCACGGTAG

The result obtained with control fibroblasts maintained with 2200 nM FA was used as a control and data obtained with the other cells and/or conditions were referred to this value and expressed as arbitrary units (AU). The data were analyzed and quantified with the program Bio-Rad CFX Maestro 2.3, Version 5.3.022.1030.

### Statistics

All data are presented as mean ± SEM. Statistical analysis was performed with GraphPad Prism 8.0.1 program. One-way ANOVA uncorrected Fisher’s LSD test was used when more than 2 groups were compared. Two-way ANOVA uncorrected Fisher’s LSD was used when comparing different conditions inside more than 2 groups (control vs LND and different cell culture conditions). If the data have Normal distribution, a paired two-tailed Student’s t test was used when only 2 groups of data were concerned.

## Results

### ZMP accumulates in LND fibroblasts cultured with a complete physiological medium (Plasmax-PV)

Recently, we have described that ZMP, an intermediary of the de novo purine biosynthetic pathway, accumulates in LND fibroblasts maintained with RPMI medium containing physiological levels of FA (López et al. 2020). However, RPMI, although being a widely used commercial medium, does not contain glucose, amino acids, and many other metabolites at physiological levels. As described before, HPLM aims to better recapitulate the physiological plasma composition. It has been reported that the uric acid present at physiological concentrations in HPLM directly inhibits uridine monophosphate synthase (UMPS), an enzyme involved in pyrimidine biosynthesis, impacting in the metabolism of cancer cells (Cantor et al. 2017). To reveal any further alterations that may have been masked by the usage of non-physiological media, we cultured fibroblasts with RPMI containing 2200 nM or 25 nM of FA, RPMI with 25 nM of FA and 350 μM uric acid (both compounds at physiological levels) or with HPLM (2200 nM FA and 350 μM uric acid). Cell extracts were obtained with 0.4 N perchloric acid (PCA) and ZMP levels were determined by High Performance Liquid Chromatography (HPLC).

**Fig. 2:**
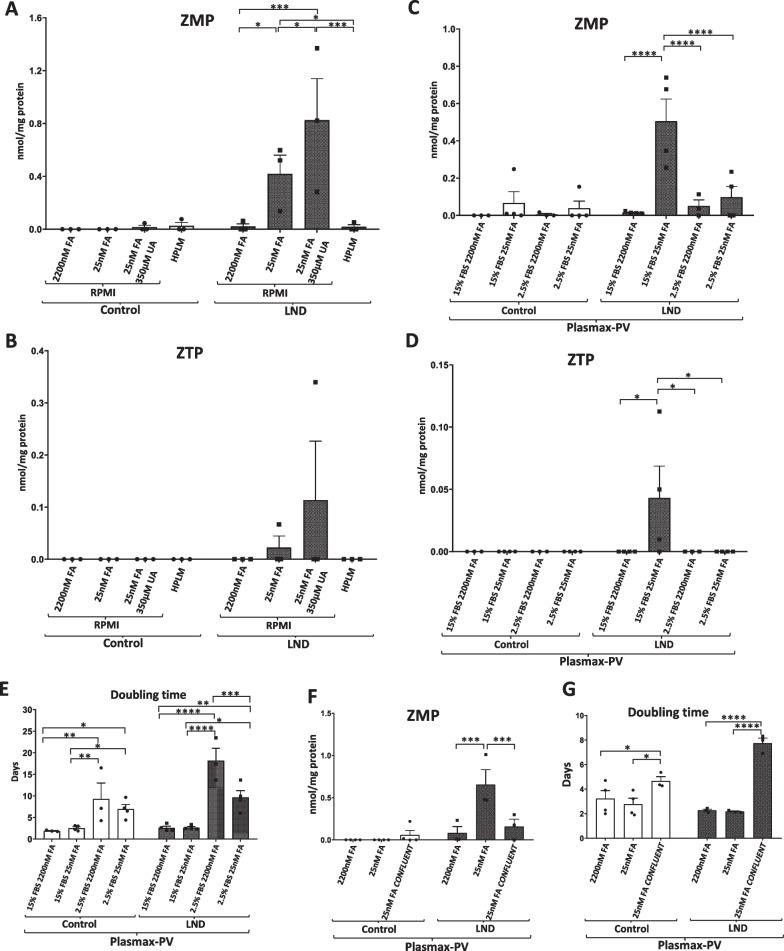
ZMP and ZTP accumulation in LND fibroblasts maintained with different culture media. **A**, **B** Fibroblasts were incubated for 5 days in RPMI medium with 2200 nM FA, 25 nM FA, 2200 nM FA + 350 μM UA or in HPLM medium. Cell extracts were obtained with 0.4 N PCA and ZMP and ZTP levels determined by HPLC. Results are expressed as nmol ZMP (**A**) or ZTP (**B**) per mg protein. **C**, **D** Fibroblasts were incubated for 7 days in Plasmax-PV medium containing 2.5% or 15% FBS and with 2200 nM or 25 nM FA. Cell extracts were obtained with 0.4 N PCA and ZMP and ZTP levels determined by HPLC. Results are expressed as nmol ZMP (**C**) or ZTP (**D**) per mg protein. **E** Doubling time was measured for fibroblasts cultivated for 7 days in each culture condition. Results are expressed as days. **F** Fibroblasts were incubated for 7 days in Plasmax-PV medium with 2200 nM or 25 nM FA and confluent fibroblasts were incubated only with Plasmax-PV containing 25 nM FA. Results are expressed as nmol ZMP per mg protein. **G** Doubling time of fibroblasts cultivated for 7 days in each culture condition. Results are expressed as days. Each graph represents the mean ± SEM of at least 3 control individuals and 3 patients with LND. *P < 0.05; P** < 0.01; P*** < 0.005 ****P < 0.001. Two-way ANOVA, Fisher’s multiple comparison test

As previously reported, LND fibroblasts cultured with RPMI containing physiological levels of FA (25 nM) accumulated ZMP (Fig. 2A) (López et al. 2020). We also detected ZTP at lower levels in one LND sample (Fig. 2B). LND fibroblasts cultivated with RPMI containing physiological levels of uric acid and FA also accumulated ZMP and ZTP (Fig. 2A, B). However, cells maintained with RPMI plus 2200 nM FA or with HPLM did not accumulate ZMP (Fig. 2A). Control fibroblast did not accumulate significant levels of ZMP in any condition (Fig. 2A). HPLM, although aims to better recapitulate blood plasma conditions, does not contain physiological levels of vitamins, including FA (Fig. 1B).

Plasmax, another commercial cell culture medium, contains physiological levels of glucose, glutamine and many metabolites, but very high levels of vitamins, similarly to HPLM (Voorde et al. 2019). Having determined how important folic acid at physiological levels is to detect ZMP accumulation in LND fibroblasts, we prepared a special Plasmax medium without vitamins and supplemented it with all the vitamins, including FA, to reach physiological levels. This final medium, Plasmax containing physiological levels of vitamins, was named Plasmax-PV (Fig. 1B, C). A complete comparison of RPMI 1640, HPLM and Plasmax-PV composition is included in Additional file 1: Figure S1.

We cultivated control and LND fibroblasts with Plasmax-PV for 7 days. To prevent exhaustion of nutrients and vitamins that are present at low levels in Plasmax-PV, the media was changed daily for the last 4 days (Gardner et al. 2022). Moreover, to analyse the influence of fetal bovine serum (FBS) concentration, some experiments were performed by supplementing the media either with 15% or 2.5% FBS.

HPLC analysis showed that LND fibroblasts cultured with Plasmax-PV containing 15% of FBS accumulate ZMP, but not when the media was supplemented with high levels of FA (Fig. 2C). ZMP accumulation was markedly decreased in media containing 2.5% of FBS (Fig. 2C). Likewise, small amounts of ZTP were detected in LND fibroblasts maintained with Plasmax-PV at 15% FBS, but not at 2.5% FBS (Fig. 2D). Control fibroblasts did not accumulate ZMP nor ZTP under any circumstance (Fig. 2C and D).

Hypoxanthine and guanine levels present in the extracellular media were quantified together since our technique does not allow their separation. As expected, LND fibroblasts cultured with RPMI or Plasmax-PV presented higher levels of hypoxanthine/guanine in the extracellular media than control fibroblasts. We also observed larger hypoxanthine and guanine levels in LND fibroblasts cultivated in Plasmax-PV (8 µM) than RPMI (5 µM) (Additional file 2: Figure S2). This difference is due to the fact that Plasmax-PV medium contains higher levels of hypoxanthine than RPMI (Fig. 1C).

### Cellular growth is necessary for the accumulation of ZMP in LND fibroblasts

As shown in Fig. 2E, Plasmax-PV containing 2.5% FBS strongly prevented cell growth in both control and LND fibroblasts. Control and LND fibroblasts cultivated with Plasmax-PV containing 15% FBS needed about 2 days to duplicate, whereas in cells maintained with 2.5% FBS the doubling time increased to about 10 days or more (Fig. 2E). As explained before, ZMP accumulation in LND fibroblasts cultured at 2.5% of FBS was markedly reduced (Fig. 2C). Indeed, preventing cell growth by using confluent cells (Fig. 2G) also reduced ZMP accumulation in LND fibroblasts (Fig. 2F).

In conclusion, both growing cells at 2.5% of FBS or seeding cells at high confluent ratios, reduced fibroblasts growth which turns out to prevent ZMP accumulation (Fig. 2C, E, F, G). Cell growth appears to be necessary for the accumulation of ZMP and this might be relevant to understand the physiopathology of LND (see Discussion). We performed all the following experiments with media containing 15% FBS to mimic in vivo conditions since LND patients have high levels of ZMP in erythrocytes (Sidi and Mitchell 1985; Ceballos-Picot et al. 2015) as well as derivates in the urine and brain (López et al. 2020).

### Plasmax-PV decreases total purine nucleotide content in human fibroblasts

**Fig. 3:**
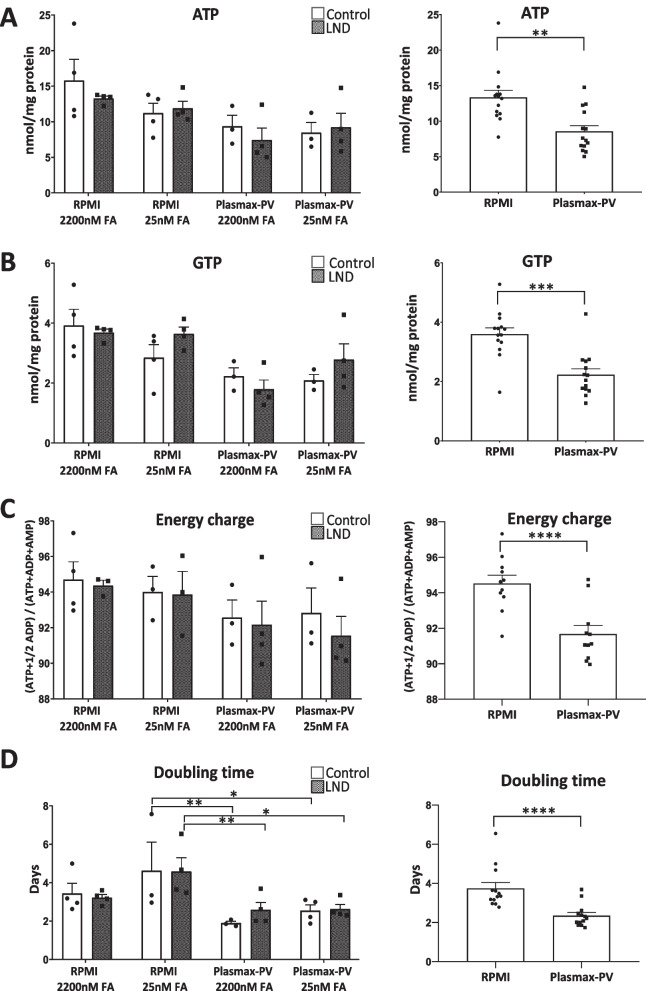
Plasmax-PV decreases the total purine nucleotide content of human fibroblasts. Fibroblasts were incubated for 7 days in Plasmax-PV or RPMI medium with 15% FBS containing 2200 nM or 25 nM FA. Cell extracts were obtained with 0.4 N PCA and purines were determined by HPLC. Graphs on the left represent the mean ± SEM of at least 3 control individuals and 3 patients with LND. Graphs on the right represent the mean ± SEM of both controls and LND patients grouped by the medium used (RPMI or Plasmax-PV). Results of ATP (**A**) and GTP (**B**) are expressed as nmol/mg protein. Adenylate energy charge is expressed as ([ATP] + 1/2[ADP]/([ATP] + [ADP] + [AMP]) (**C**) and doubling times as days (**D**). Graphs on the left are analysed with two-way ANOVA, Fisher’s multiple comparison test and graphs on the right with a paired t-student test. P* < 0.05; P** < 0.01; P*** < 0.001; P**** < 0.0001

The most abundant purine nucleotides were determined by HPLC. There were no differences in ATP and GTP levels between control and LND fibroblasts maintained either with RPMI or Plasmax-PV at different FA concentrations (Fig. 3A, B, left panels). The adenylate energy charge, defined as the ratio ([ATP] + 1/2 [ADP])/([ATP] + [ADP] + [AMP]) by Atkinson and Walton (Atkinson and Walton 1967), was similar in controls and LND fibroblasts (Fig. 3C, left panel). However, the fibroblasts maintained with Plasmax-PV presented lower levels of ATP, GTP, and adenylate energy charge, that the ones cultured with RPMI. When data were grouped by the medium used (RPMI or Plasmax-PV) there was a significant reduction of ATP, GTP, and adenylate energy charge in the fibroblasts maintained with Plasmax-PV (Fig. 3A–C, right panels). Despite this decrease, fibroblasts grew well in Plasmax-PV and, in fact, had a lower doubling time (Fig. 3D). Decreased levels of ATP in cancer cell lines maintained with Plasmax have been reported before, without affecting proliferation rates (Hennequart et al. 2021). Moreover, Plasmax increases the number of colonies formed by cancer cell lines (Voorde et al. 2019). Since Plasmax-PV better recapitulates physiological conditions, all the following experiments were performed with this medium.

### ZMP accumulation does not induce ADSL inhibition or AMPK activation in LND fibroblasts

Since ZMP is an inhibitor of ADSL (Sabina et al. 1982, 1985), we analysed the levels of succinyl-ZMP and succinyl-AMP (substrates of ADSL) in PCA extracts obtained from control and LND fibroblasts, and the nucleosides (succinyl-AICAr and succinyl-adenosine) in the extracellular media. In addition, succinyl-AICAr and succinyl-adenosine were also quantified in the urines collected from control patients, LND variants (HRH and HND) and one patient with ADSL deficiency as a positive control.

**Fig. 4:**
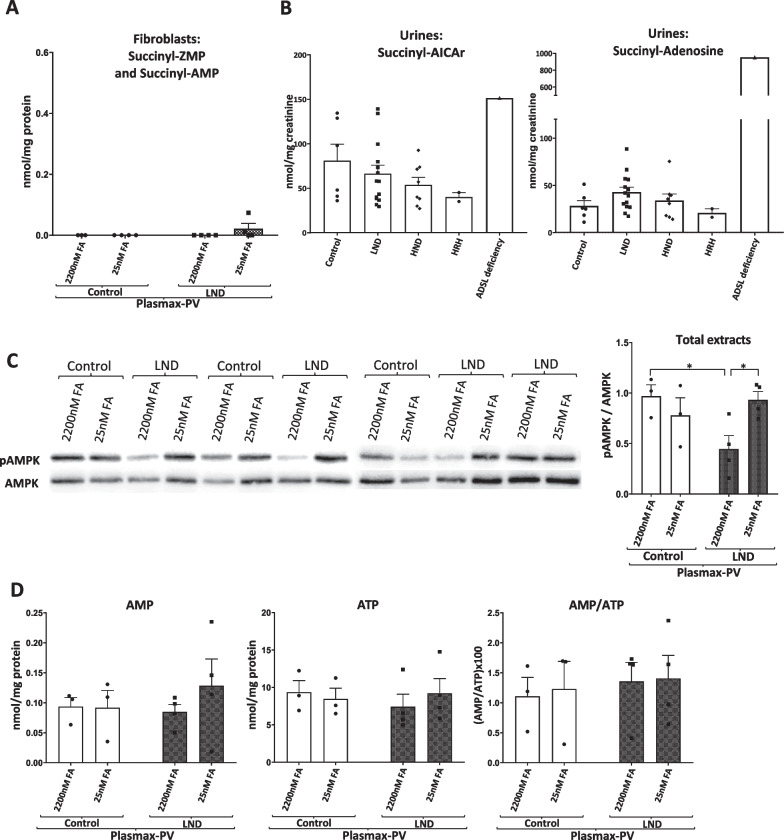
Succinyl-metabolites do not accumulate in fibroblasts and urines from LND patients, and AMPK is not activated in LND fibroblasts. **A** Fibroblasts were incubated for 7 days in Plasmax-PV medium with 15% FBS containing 2200 nM or 25 nM FA. Cell extracts were obtained with 0.4 N PCA and succinyl-AICAR/succinyl-AMP levels determined by HPLC. Graphs represent the mean ± SEM of at least 3 control individuals and 3 patients with LND, expressing the results as nmol/mg protein. Two-way ANOVA, Fisher’s multiple comparison test shows no significant differences. **B** PCA metabolites extraction was performed in urines from control individuals (N = 6), patients with LND (N = 14), HND (N = 8), HRH (N = 2), or ADSL deficiency (N = 1). Succinyl-AICAr and succinyl-adenosine levels were determined by HPLC. Results are expressed as nmol/mg creatinine. Graphs represent the mean ± SEM. One-way ANOVA, Fisher’s multiple comparison test shows no statistical differences. ADSL deficiency urine sample has not been included in the statistical analysis. **C** AMPK activity determined by Western blot, as the ratio pAMPK/AMPK, in total cell extracts from control and LND fibroblasts cultivated with Plasmax-PV containing different levels of FA. 80 µg of protein were loaded per well. The results are the mean ± SEM of 3 controls and 4 LND patients. *P < 0.05. Two-way ANOVA, Fisher’s multiple comparison test. **D** Fibroblasts were incubated for 7 days in Plasmax-PV medium with 15% FBS containing 2200 nM or 25 nM FA. Cell extracts were obtained with 0.4 N PCA and AMP and ATP levels determined by HPLC. Results of AMP and ATP expressed as nmol/mg protein, and the AMP/ATP ratio is the mean ± SEM of 3 control individuals and 4 patients with LND. A two-way ANOVA shows no significant differences

We did not detect succinyl-adenosine or succinyl-AICAr in the extracellular medium of control and LND fibroblasts. Very low levels of succinyl-ZMP/succinyl AMP were detected in PCA extracts obtained from both control and LND fibroblasts (Fig. 4A). Succinyl-ZMP was quantified together with succinyl-AMP since our HPLC methodology does not allow separation of these compounds. We did not observe any increase in the succinyl metabolites, neither succinyl-adenosine nor succinyl-AICAr in the urine obtained from LND patients or its variants HND and HRH (Fig. 4B). A urine sample obtained from a patient with ADSL deficiency was used as a positive control, showing high levels of succinyl-AICAr and very high levels of succinyl-adenosine compared with control values (Fig. 4B). As previously described (López et al. 2020), AICA and AICAr, which are derivatives from ZMP, were increased in urines from LND patients and its variants compared to control individuals (Additional file 3: Figure S3A, B). These results suggest that ADSL is not significantly inhibited in LND patients.

Since ZMP is well known to mimic AMP effects on AMPK activation by a direct allosteric regulation leading to AMPK phosphorylation (Fig. 1A) (Corton et al. 1995), we evaluated AMPK activity. For that, we performed Western blot experiments measuring the pAMPK/AMPK ratio in total cell extracts from control and LND fibroblasts cultivated with Plasmax-PV containing different FA levels. As shown in Fig. 4C, there was a decrease in pAMPK/AMPK ratio in LND fibroblasts cultivated with high levels of folate. However, AMPK was not activated in LND fibroblasts maintained with Plasmax-PV and physiological levels (25 nM) of FA compared to controls. This indicates that ZMP accumulation in LND fibroblasts cultivated with Plasmax-PV does not induce AMPK activation. Moreover, we did not observe significant differences in the AMP/ATP ratio between LND and control fibroblasts under any culture condition (Fig. 4D).

Aiming to increase purine synthesis efficiency, enzymes involved in the de novo purine biosynthesis pathway are compartmentalized into a complex known as purinosome that localizes close to the mitochondria (Pedley et al. 2022). Therefore, ZMP produced during the de novo purine biosynthesis in LND fibroblasts may not spread homogenously around the cell, but higher levels may be present around the mitochondria. Although AMPK is considered a cytosolic enzyme, it can also be in the outer mitochondrial membrane (Drake et al. 2021), therefore, ZMP could activate mitochondrial AMPK. As shown in Additional file 4: Figure S4, AMPK activity, measured as pAMPK/AMPK ratio, was higher in the mitochondrial than in the cytoplasmatic fractions, but no differences were observed between control and LND fibroblasts cultivated with Plasmax-PV.

Altogether, our results suggest that ADSL is not inhibited, and AMPK is not activated in LND fibroblasts that accumulate ZMP at physiological levels of FA. The intracellular ZMP levels attained (200 µM, calculated from the cell volume of fibroblasts) although being four times higher than intracellular AMP (50 µM) do not have any effect on ADSL and AMPK activity.

### mTOR activity is not altered in LND fibroblasts maintained under physiological culture conditions

**Fig. 5:**
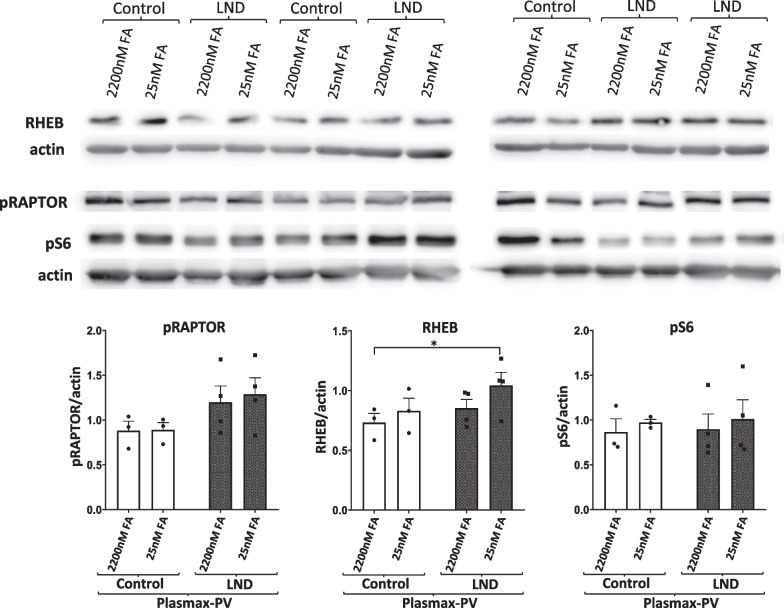
mTORC1 activity is not altered in LND fibroblasts cultivated with Plasmax-PV. RHEB (mTORC1 activator), pRAPTOR (mTORC1 inhibitor) and the mTORC1 downstream target pS6 were determined by Western blot in total cells extracts prepared from control and LND fibroblasts cultivated with Plasmax-PV containing different levels of FA. 80 µg of protein were loaded per well. Actin was used for normalization and the results are the mean ± SEM of 3 controls and 4 LND patients. *P < 0.05. Two-way ANOVA, Fisher’s multiple comparison test

The mammalian target of rapamycin (mTOR) pathway signals cells to grow when the availability of nutrients and the energy status of the cell is favourable. It has been reported that mTORC1 pathway is inhibited in HGprt deficient midbrains dopaminergic neuronal progenitor cells (Bell et al. 2021). To reveal any differences in the status of mTOR pathway between control and LND fibroblasts we evaluated levels of mTORC1 related proteins by Western blot: the mTORC1 activator RHEB, the mTORC1 inhibitor RAPTOR (pRAPTOR), and the mTORC1 effector ribosomal protein 6 (pS6) in total protein extracts from control and LND fibroblasts cultivated with Plasmax-PV containing different FA levels. We only found a slightly significant increase in RHEB levels of LND fibroblasts cultivated with physiological levels of FA but it did not translate into increased levels of the mTORC1 effector protein pS6 (Fig. 5).

ATF4 is a nutrient sensing transcription factor that respond to amino acid deprivation and endoplasmic reticulum stress. It was described to be activated in cancer cell lines cultivated with Plasmax when the media was not properly changed every 24 h (Hennequart et al. 2021; Golikov et al. 2022b). As explained by Gardner et al., Plasmax medium, if not changed in a daily manner, might be susceptible of nutrient depletion leading to cellular stress (Gardner et al. 2022). We did not detect any increase of ATF4 levels (Additional file 5: Figure S5A) or changes in mTORC1 activity (Additional file 5: Figure S5B) in fibroblasts cultivated with Plasmax-PV in comparison to commercial media. This indicates that our experimental culture conditions with Plasmax-PV are satisfying the cell demands, not leading to nutrient exhaustion and stress situations.

### LND fibroblasts maintained in Plasmax-PV have decreased mitochondrial membrane potential and increased glycolytic capacity than controls

Next, we analyzed the mitochondrial functionality under conditions where fibroblasts accumulate ZMP, since it was previously described that ZMP directly inhibits the mitochondrial respiratory complex I in rat hepatocytes (Guigas et al. 2007). Mitochondrial respiratory complex I is also described to be inhibited in the brain of HGprt-deficient mice (Vinokurov et al. 2023).

**Fig. 6:**
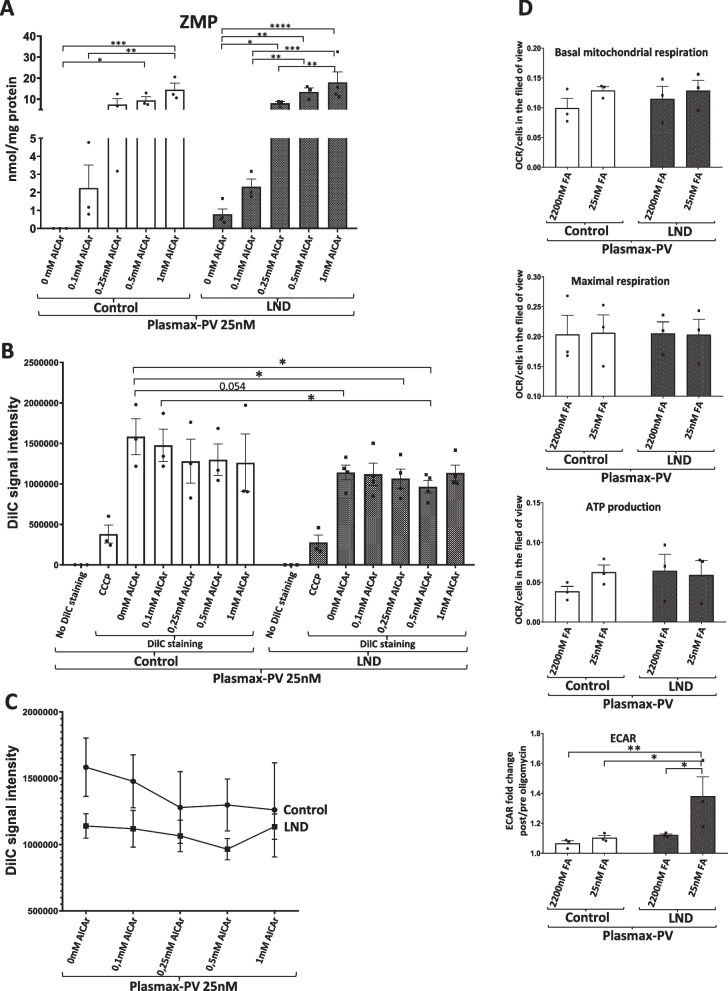
Metabolic alterations in LND fibroblasts under physiological conditions. **A** ZMP levels in fibroblasts treated with AICAr. Fibroblasts were maintained with Plasmax-PV medium containing 25 nM FA for 7 days and treated with or without AICAr at different concentrations (0.1 mM, 0.25 mM, 0.5 mM, and 1 mM) for 24 h. Cell extracts were obtained with 0.4 N PCA and ZMP levels determined by HPLC, and results expressed as nmol/mg protein. **B**, **C** Mitochondrial potential is decreased in LND fibroblasts. Cells were incubated with DilC or DilC plus CCCP and signal intensity was quantified by cytometry. The results are the mean ± SEM of 3 controls and 4 LND patients. A two-way ANOVA shows significant differences with P < 0.05 between control and LND groups. *P < 0.05; P** < 0.01; P*** < 0.005 ****P < 0.001. Two-way ANOVA, Fisher’s multiple comparison test. **D** Mitochondrial respiration was evaluated in control and LND fibroblasts cultivated with Plasmax-PV containing different levels of FA by seahorse assay as described in Material and Methods. Basal mitochondrial respiration, maximal respiration and ATP production are expressed as the oxygen consumption rate (pmol/min) normalized by the number of cells in the field of view. Analysis or the extracellular acidification rate (ECAR) is expressed as the fold change after oligomycin treatment. The results are the mean ± SEM of 3 controls and 3 LND patients. *P < 0.05; **P < 0.01. Two-way ANOVA, Fisher’s multiple comparison test

We cultivated fibroblasts with Plasmax-PV containing physiological levels (25 nM) of FA and treated them for 24 h with increased amounts (0, 0.1, 0.25, 0.5 and 1 mM) of AICA ribonucleoside (AICAr). AICAr is taken inside the cell and metabolized into ZMP by adenosine kinase (Sabina et al. 1985). ZMP quantification by HPLC showed that, as expected, only LND fibroblasts, but not the controls, accumulated ZMP under physiological levels of FA. When the fibroblasts were treated with 0.1 mM AICAr, ZMP accumulated at similar levels in both control and LND fibroblasts and reached out its maximum with 0.25 mM AICAr treatment (Fig. 6A).

We quantified the mitochondrial membrane potential, measured as DilC signal by cell cytometry, in control and LND fibroblasts treated with AICAr. Cells incubated with the membrane disrupter CCCP served as a positive control of mitochondrial depolarization. Non treated LND fibroblasts showed lower DilC signal than controls (P = 0.054) (Fig. 6B). When LND fibroblasts were treated with AICAr this difference was clearer (P < 0.05) and disappeared when comparing control and LND fibroblasts treated with the same AICAr concentration (Fig. 6B). DilC levels tend to decrease in control, but not in LND fibroblast, upon higher doses of AICAr (Fig. 6C), suggesting that ZMP accumulation may lead to decrease mitochondrial membrane potential.

Seahorse analysis showed no differences in the basal mitochondrial respiration, maximal respiration capacity and ATP production between control and LND fibroblasts cultivated with Plasmax-PV and different FA levels (Fig. 6D). However, analysis of the extracellular acidification rate (ECAR) after oligomycin treatment showed significant differences between control and LND fibroblasts. ECAR fold change was higher in LND fibroblasts than controls when cultivated with Plasmax-PV at physiological levels (25 nM) of FA (Fig. 6D, bottom). These results suggest that LND fibroblasts have an increased glycolytic capacity probably as a metabolic adaptation to supply precursors for the de novo purine biosynthesis. We did not observe any difference in the basal mitochondrial respiration, maximal respiration capacity and ATP production between fibroblasts cultivated with RPMI or Plasmax-PV at physiological levels of FA (Additional file 6: Figure S6). Notably, ECAR differences between control and LND fibroblasts maintained with 25 nM FA were only observed in Plasmax-PV but not in RPMI (Additional file 6: Figure S6), highlighting the importance of using media that most meticulously recapitulate the physiological scenario.

### LND fibroblasts maintained under physiological conditions show increased expression of the folate transporter SLC19A1

**Fig. 7:**
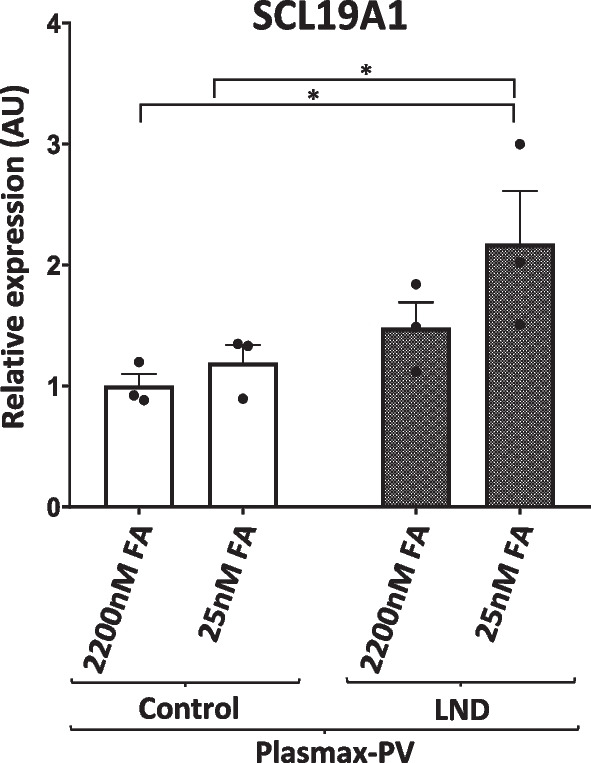
LND fibroblasts express higher levels of the folate carrier *SLC19A1*. Control and LND fibroblasts were cultured in Plasmax-PV containing different levels of FA. Total RNA was extracted and *SLC19A1* mRNA levels quantified by qRT-PCR as described in Material and Methods. Results were expressed in arbitrary units (AU), using control fibroblasts maintained with 2200 nM FA as a reference condition for normalization of the data. The results are the mean ± SEM of 3 controls and 4 LND patients. *P < 0.05. Two-way ANOVA, Fisher’s multiple comparison test

Folic acid, in the form of 10-formyltetrahydrofolate, is a crucial intermediate in the de novo purine synthesis pathway (Fig. 1A). Mammals cannot synthesize folates; they strongly depend on their uptake from the extracellular environment. Folate uptake is mediated by three types of folate transporters: the reduced folate carrier (RFC), proton-coupled folate transporter (PCFT), and folate receptors (FRs) being RFC ubiquitously expressed and used in most tissues (Hou and Matherly 2014). We quantified the expression of SLC19A1 mRNA, which encodes the RFC, in control and LND fibroblasts cultivated with Plasmax-PV containing physiological (25 nM) or high levels (2200 nM) of FA. LND fibroblasts cultivated with 25 nM FA showed higher SLC19A1 expression than controls, suggesting a metabolic adaptation to keep the high demand of folic acid in the de novo purine biosynthetic pathway when the salvage pathway is deficient (Fig. 7).

### Reduced cell migration in LND fibroblasts maintained with Plasmax-PV

**Fig. 8:**
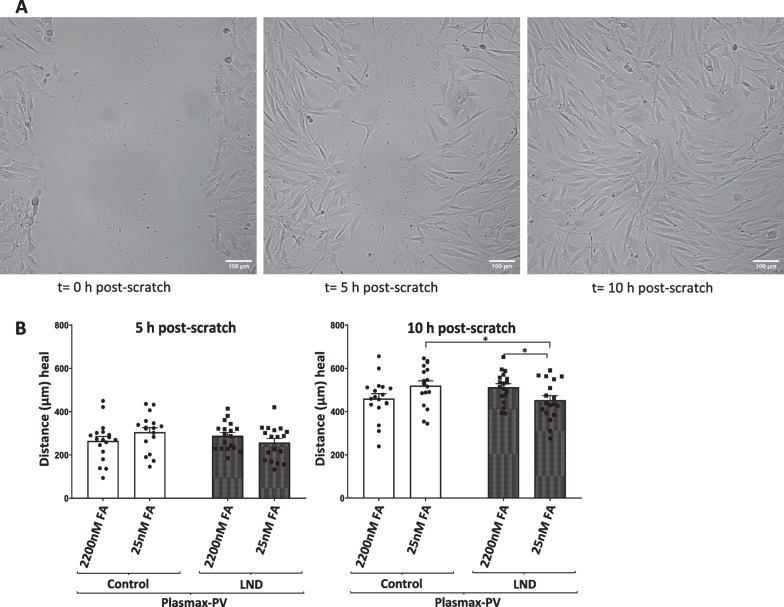
LND fibroblasts under physiological conditions show reduced migratory capacity. Migratory capacity was determined by scratch assay, as described in Material and methods, in control and LND fibroblasts cultivated with Plasmax-PV containing different levels of FA. **A** Images of the cell culture taken at 0, 5 and 10 h after the scratch was done. **B** Results were expressed in μm as the distance healed at each specific time point (5 and 10 h post-scratch). The results are the mean ± SEM of 3 controls and 3 LND patients taking 6 independent positions for each condition. *P < 0.05. Two-way ANOVA, Fisher’s multiple comparison test

Finally, we assessed a functional alteration in LND fibroblasts with the scratch assay, an in vitro technique to evaluate the cell migration capacity (Liang et al. 2007). An artificial gap, called “scratch”, was made on fibroblasts monolayer culture. Once the scratch was created, cells were incubated with Plasmax-PV media containing 2.5% FBS to stop cell growth and only evaluate cell migration. Images of the cell culture were taken at different times (Fig. 8A). Cell migration was measured at 5 and 10 h post-scratch showing a slight decrease in the length of healed distance at 10 h post-scratch in LND fibroblasts cultivated with 25 nM of FA compared to controls (Fig. 8B). This result suggests that LND fibroblasts have a reduced cell migration.

## Discussion

### Determining specific cell culture settings that best recapitulate physiological conditions to study Lesch Nyhan disease in primary skin fibroblasts

Widely used commercial media were not formulated aiming to best reproduce the physiological cellular environment but to avoid nutrient exhaustion to successfully cultivate cells. Because of that, the usage of HPLM and Plasmax media, that contain major components at plasma levels, have gained interest in the last years by showing striking improvement when using cancer cell models. For example, Cantor reported that tumoral cells maintained in HPLM showed decreased pyrimidine biosynthesis due to the presence of uric acid, the final catabolite of purine metabolism, in the medium (Cantor et al. 2017). Normalization of the urea cycle and the hypoxia-inducible factor (HIF) signaling, appropriate regulation of serine biosynthesis, increase colony formation capacity and effects on the energy metabolism have also been associated to the usage of physiological media with cancer cells (Hennequart et al. 2021; Voorde et al. 2019; Moradi et al. 2021). Interestingly, it has also been described that Plasmax and HPLM media may have different effects depending on the specific cell line as a reflection of the different metabolic background of each of them (Cantor et al. 2017; Golikov et al. 2022b).

Until now, the effort of using a more physiological media has been focused on cancer cells. It is still not well characterized the benefits and disadvantages of culturing other cellular models such as stem cells, iPSCs, and primary cell lines with these media. Therefore, we were not aware of how the usage of these media may affect human primary fibroblasts and which were the advantages that may provide to the study of a metabolic disease.

Of great importance, our results suggest that the usage of current commercial Plasmax and HPLM media do not recapitulate well enough the physiological alterations detected in LND patients. Vitamins are not considered major plasma components, since its concentrations are lower than 2 µM, but they are essential intermediates in the primary metabolic pathways of the cell. We only observed ZMP and ZTP accumulation in Lesch-Nyhan fibroblasts when folic acid was present at physiological levels in the cell culture (Fig. 2A–D). Therefore, to study the LND pathology, where folic acid plays a major role in keeping the de novo purine synthesis pathway, we believe Plasmax containing physiological levels of vitamins (Plasmax-PV) is a more suitable medium.

Primary fibroblasts were not able to properly grow when cultured with media containing only 2.5% of FBS and experiments with confluent cells reaffirmed that growth is key to observe the ZMP accumulation phenotype in LND fibroblasts (Fig. 2C–G). Hence, we made use of Plasmax-PV media containing 15% of FBS for further experiments to closer reproduce the in vivo scenario. Interestingly, we have found a significant decrease of ATP and GTP levels when the fibroblasts were maintained with Plasma-PV instead of RPMI medium (Fig. 3A–C), which may have an impact when studying cell metabolism. Despite this decrease in total purine content, the fibroblasts grew well. In fact, the doubling time was significantly decreased (Fig. 3D). Similar effects have been reported before in several cell lines maintained with Plasmax (Voorde et al. 2019; Hennequart et al. 2021). This could be relevant to understand LND physiopathology, since recently it has been reported that *HPRT1* knock out mice present neurodevelopmental alterations during embryogenesis, affecting proliferation and migration of cells. Specifically, proliferation of developing midbrain dopamine (mDA neurons) was increased in the mutant embryo, accompanied with a deviation from their migratory route (Witteveen et al. 2022).

Several reports- have described that the usage of Plasmax media for culturing cancer cell lines may lead to an increase activity of the stress response transcription factor ATF4 (Hennequart et al. 2021; Golikov et al. 2022a, b). Gardner et al. claimed the importance of changing Plasmax media in a daily manner to never decrease nutrient concentrations below physiological levels, thus preventing a starving situation for cells that would cause ATF4 activation and compromise mTORC1 activity. Our results clearly show that daily changes of Plasmax-PV avoid ATF4 activation and does not modify mTORC1 activity (Additional file 5: Figure S5).

This work highlights the importance of carefully evaluating which are the most suitable cell culture conditions prior to conducting experimental work in vitro. The lack of cell culture conditions that closely mimic the in vivo situation may lead to erroneous conclusions. In fact, the study of LND over the last years has been characterized by the difficulty to find important functional alterations in the HGprt deficient cells. Here, we propose cell culture conditions that may provide new insights into the biochemical and cellular alterations that occur in LND: (1) to use Plasmax media containing physiological levels of vitamins (Plasmax-PV), (2) to allow cells to properly grow by using 15% of FBS and not seeding confluent cells, and (3) to change the cell culture media in a daily manner to prevent nutrient starvation. These are general rules appropriate for studies with primary skin fibroblasts, that might need some modifications depending on the specific cell type considered. Of course, the cellular model could be improved by incubating the cells at physiological concentrations of oxygen (Moradi et al. 2021), using 3D cultures or adding physiological levels of lipids and other components not included in Plasmax-PV (Golikov et al. 2022a). Since there are not suitable in vivo animal models that reproduce LND pathophysiology (Micheli et al. 2019), improvements in the study of cells derived from the patients are necessary.

### LND fibroblasts show metabolic adaptations and functional alterations that are reversible when cultivated with high doses of folic acid

By cultivating control and LND fibroblasts with Plasmax-PV we have characterized intriguing metabolic adaptations. Due to a deficient salvage pathway for the synthesis of purines, LND fibroblasts rely on the de novo pathway which depends on the folic acid uptake from the extracellular media (Fig. 1A). LND fibroblasts cultivated with Plasmax-PV and physiological concentrations of folic acid show an increased expression of the folate carrier SLC19A1 that may favour the effectively uptake of folic acid from the media (Fig. 7). Interestingly, SLC19A1 transporter is also described to internalize cyclic dinucleotides GMP-AMP (cGAMP) that acts as a second messenger activating the endoplasmic-reticulum-resident receptor STING (stimulator of interferon genes) (Luteijn et al. 2019). The cGAMP-STING pathway is mainly involved in antiviral cellular response, but it is also related to inflammation, loss of dopaminergic neurons and neurodegenerative phenotypes (Sliter et al. 2018; Decout et al. 2021). It would be interesting to study if HGprt deficient dopaminergic neurons have a higher expression of SLC19A1 and a possible relation with the neurological disfunctions observed in the patients. Moreover, the increased glycolytic capacity of LND fibroblasts (Fig. 6D) suggests an adaption where glucose metabolites are redirected into the de novo nucleotide biosynthetic pathway. Further studies are needed to test this hypothesis.

Despite the metabolic adaptations observed in LND fibroblasts cultivated with Plasmax-PV, physiological levels of folic acid appear to limit their high demand of the de novo pathway since it leads to the accumulation of ZMP and ZTP (Fig. 2C, D). Under these conditions we have also observed some functional alterations in LND fibroblasts, such a modest decrease in their migration capacity (Fig. 8) and a drop in their mitochondrial membrane potential compared to control fibroblasts (Fig. 6B, C). This decrease in the mitochondrial membrane potential is also detected in control fibroblasts treated with AICAr (Fig. 6B, C), suggesting a possible correlation between ZMP accumulation and mitochondrial alterations, as previously reported in the literature (Guigas et al. 2007).

Of great importance for possible therapeutic approaches, the metabolic adaptations and functional alterations detected are rescued when LND fibroblasts are cultivated with high concentrations of folic acid (2200 nM) (Fig. 2C, D, 6D, 7, 8B). This result raises the question regarding whether LND should be treated with folate supplements, a treatment that may need to begin at a very early age.

On the other side, ZMP accumulation does not induce ADSL inhibition (Fig. 4A and B) or AMPK activation (Fig. 4C, D, and Additional file 4: Figure S4), and the mTORC1 activity is not altered in LND fibroblasts (Fig. 5). However, we should be cautious when discarding these targets since in the literature there are examples of functional alterations that are cell type specific. For example, it has been reported that HGprt deficiency leads to a decrease mTORC1 activity in midbrain dopaminergic neuronal progenitor cells but not in cortical neuronal progenitor cells or iPSCs (Bell et al. 2021).

Overall, this study highlights the importance of taking a closer look at the cell culture conditions to reveal metabolic and functional alterations that may have not been depicted due to using non-physiological culture conditions and that could be of great interest for the development of promising therapeutic approaches.

## Conclusions

Standard cell culture media are not optimal for the study of Lesch Nyhan disease (LND). We set up a new medium containing physiological levels of vitamins (Plasmax-PV) that reveals new cellular alterations in LND masked by the usage of non-physiological media. LND fibroblasts maintained with Plasmax-PV present higher expression of the folate carrier SCL19A1, increased glycolytic capacity, decreased mitochondrial potential, and impaired migration rate compared with controls. Importantly, the biochemical and cellular alterations detected in LND fibroblasts can be reverted with high levels of folic acid, suggesting a potential treatment for LND. Plasmax-PV gives insights into the pathogenesis of LND and might be useful for other metabolic disorders.

### Supplementary Information

**Additional file 1: Figure S1.** Formulations of Roswell Park Memorial Institute (RPMI) medium 1640, Human Plasma-Like Medium (HPLM) and Plasmax with physiological vitamins (Plasmax-PV). All the concentrations are reported in μM. NA = not available.

**Additional file 2: Figure S2.** LND fibroblasts maintained with RPMI or Plasmax-PV accumulate hypoxanthine. Extracellular medium from fibroblasts cultivated for 7 days in the specified media conditions was collected, filtered, and the levels of hypoxanthine/guanine measured by HPLC. Graphs represent the mean ± SEM of at least 3 control individuals and 3 patients with LND, expressing the results as μM. Two-way ANOVA shows significant differences with P < 0.0001 between control and LND groups. *P < 0.05. Two-way ANOVA, Fisher’s multiple comparison test.

**Additional file 3: Figure S3.** AICA and AICAr quantification in urines. PCA metabolites extraction was performed in urines from control individuals (N = 6), patients with LND (N = 14), HND (N = 8), HRH (N = 2), and ADSL deficiency (N = 1). AICA (A) and AICAr (B) levels were determined by HPLC, and the results expressed as nmol/mg creatinine. Graphs represent the mean ± SEM. *P < 0.05 **P < 0.01. One-way ANOVA, Fisher’s multiple comparison test.

**Additional file 4: Figure S4.** Mitochondrial and cytoplasmatic AMPK activity is similar in control and LND fibroblasts. pAMPK and AMPK levels were determined by Western blot in cytoplasmatic and mitochondrial fractions obtained from control and LND fibroblasts cultivated with Plasmax-PV containing different levels of FA. Voltage-dependent anion channel (VDAC) was used as a mitochondrial marker and the hypoxanthine–guanine phosphoribosyltransferase enzyme (HGprt) as a cytosolic marker present only in the cytoplasm of control fibroblasts but not in LND. AMPK activity expressed as the ratio pAMPK/AMPK is represented in the graph. The results are the mean ± SEM of 4 controls and 4 LND patients. 30 µg of protein were loaded per well. Two-way ANOVA, Fisher’s multiple comparison test shows no significant differences.

**Additional file 5: Figure S5.** Plasmax-PV does not increase ATF4 expression and does not alter mTORC1 activity compared with RPMI. Fibroblasts were incubated for 7 days in Plasmax-PV or RPMI medium with 15% FBS containing 25 nM FA. Total cell extracts were obtained, and the expression of ATF4 (A) and the mTORC1 related proteins RHEB, pRAPTOR and pS6 (B) were determined by Western blot, quantified, and normalized by actin levels. 80 µg of protein were loaded per well. The graph represents the mean ± SEM of 4 individuals (2 controls and 2 patients with LND). Paired t-test shows no significant differences.

**Additional file 6: Figure S6.** Oxygen consumption rate (OCR) is similar in fibroblasts maintained with RPMI and Plasmax, but not glycolytic capacity in LND fibroblasts. Mitochondrial respiration was evaluated in control and LND fibroblasts cultivated with RPMI or Plasmax-PV containing physiological levels (25 nM) of FA by seahorse assay as described in Material and Methods. Basal mitochondrial respiration, maximal respiration and ATP production are expressed as the oxygen consumption rate (pmol/min) normalized by the number of cells in the field of view. The results are the mean ± SEM of 6 individuals (3 controls and 3 LND). A paired t-student test shows no significant differences. Extracellular acidification rate (ECAR), expressed as the fold change after oligomycin treatment, is increased in LND fibroblasts maintained with Plasmax-PV. The results are the mean ± SEM of 3 controls and 3 LND patients. *P < 0.05. Fisher’s multiple comparison test.

## Data Availability

Datasets used and analysis during the current study are available from the corresponding author on reasonable request.

## Abbreviations

AICAr: 5-Aminoimidazole-4-carboxamide ribonucleoside
FA: Folic acid
LND: Lesch-Nyhan disease
PCA: Perchloric acid
ZMP/AICAR: 5-Aminoimidazole-4-carboxamide riboside 5ʹmonophosphate
