# Supplementary material for: A new physiological medium uncovers biochemical and cellular alterations in Lesch-Nyhan disease fibroblasts
Source: Mol Med. 2024 Jan 3;30:3. doi: 10.1186/s10020-023-00774-8 (PMC10765874; doi:10.1186/s10020-023-00774-8)
Supplement: Supplementary file 1 — Additional file 1: Figure S1. Formulations of Roswell Park Memorial Institute (RPMI) medium 1640, Human Plasma-Like Medium (HPLM) and Plasmax with physiological vitamins (Plasmax-PV). All the concentrations are reported in μM. NA = not available. [file 10020_2023_774_MOESM1_ESM.pdf]

	RPMI 1640	HPLM	Plasmax-PV
**Proteogenic amino acids (µM)**			
L-Alanine	NA	430	510
L-Arginine	1149	110	64
L-Asparagine	379	50	41
L-Aspartic acid	150	20	6
L-Cysteine	NA	40	33
L-Glutamate	136	80	98
L-Glutamine	2055	550	650
Glycine	133	300	330
L-Histidine	97	110	120
L-Isoleucine	382	160	170
L-Leucine	382	70	140
L-Lysine	219	200	220
L-Methionine	101	30	30
L-Phenylalanine	91	80	68
L-Proline	174	200	360
L-Serine	286	150	140
L-Threonine	168	140	240
L-Tryptophan	25	60	78
L-Tyrosine	111	80	74
L-Valine	171	220	230

	RPMI 1640	HPLM	Plasmax-PV
**Non-proteogenic amino acids (µM)**			
α-Aminobutyrate	NA	20	41
L-Citruline	NA	40	55
L-Cystine	208	100	65
L-Homocysteine	NA	NA	9
4-Hydroxy-L-proline	153	20	13
L-Ornitine	NA	70	80
L-Pyroglutamate	NA	NA	20

	RPMI 1640	HPLM	Plasmax-PV
**Amino acids derivatives (µM)**			
L-Acetyl glycine	NA	90	70
L-Carnosine	NA	NA	6
Glutathione (reduced)	3	25	37
Taurine	NA	90	130
Betaine	NA	70	72

	RPMI 1640	HPLM	Plasmax-PV
**Other components (µM)**			
Acetate	NA	40	42
Acetone	NA	60	55
Acetyl carnitine	NA	NA	5
Citrate	NA	130	114
Carnitine	NA	40	46
Creatine	NA	40	37
Creatinine	NA	75	74
Formate	NA	50	33
Fructose	NA	40	NA
Galactose	NA	60	NA
D-Glucose	11101	5000	5560
Glycerol	NA	120	82
2-Hydroxybutyrate	NA	50	31
3-Hydroxybutyrate	NA	50	77
3-Hydroxyisobutyrate	NA	NA	20
Hypoxanthine	NA	10	5
Malonate	NA	10	NA
Lonolei acid	NA	NA	NA
Lipoic acid	NA	NA	NA
Methyl acetoacetate	NA	NA	41
Phenol red	13.3	14	25
Pyruvate	NA	50	100

	RPMI 1640	HPLM	Plasmax-PV
**Inorganic salts (µM)**			
Ammonium chloride	NA	40	50
Calcium chloride	NA	2350	1800
Calcium nitrate	424	40	NA
Magnesium chloride	NA	480	NA
Magnesium sulfate	407	350	813
Potassium chloride	5333	4100	5330
Potassium nitrate	NA	NA	NA
Sodium bicarbonate	23810	24000	26191
Sodium chloride	103448	106000	118706
Sodium phosphate monobasic	NA	NA	1010
Sodium phosphate dibasic	5634	951	NA

	RPMI 1640	HPLM	Plasmax-PV
**Trace elements (µM)**			
Ammonium metavanadate	NA	NA	0.0026
Cupric sulfate	NA	NA	0.0052
Ferrich chloride	NA	NA	NA
Ferric nitrate	NA	NA	0.1238
Ferric sulfate	NA	NA	1.0428
Manganous chloride	NA	NA	0.0002
Sodium selenite	NA	NA	0.0289
Zinc sulfate	NA	NA	1.5
