# Supplementary figures and images for: A new physiological medium uncovers biochemical and cellular alterations in Lesch-Nyhan disease fibroblasts
Source: Mol Med. 2024 Jan 3;30:3. doi: 10.1186/s10020-023-00774-8 (PMC10765874; doi:10.1186/s10020-023-00774-8)

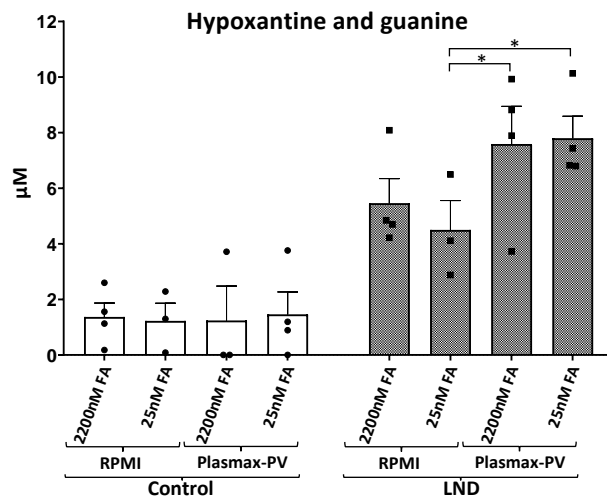

Supplement: Supplementary file 2 — Additional file 2: Figure S2. LND fibroblasts maintained with RPMI or Plasmax-PV accumulate hypoxanthine. Extracellular medium from fibroblasts cultivated for 7 days in the specified media conditions was collected, filtered, and the levels of hypoxanthine/guanine measured by HPLC. Graphs represent the mean ± SEM of at least 3 control individuals and 3 patients with LND, expressing the results as μM. Two-way ANOVA shows significant differences with P < 0.0001 between control and LND groups. *P < 0.05. Two-way ANOVA, Fisher’s multiple comparison test. [file 10020_2023_774_MOESM2_ESM.pdf]

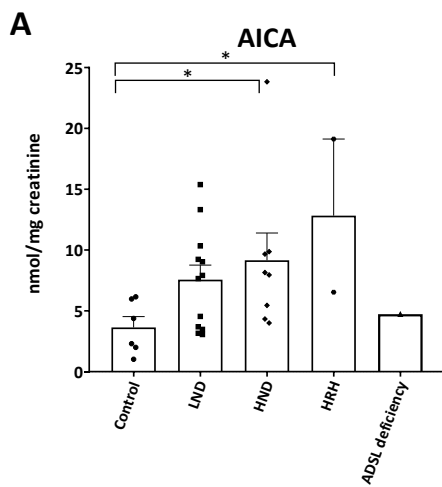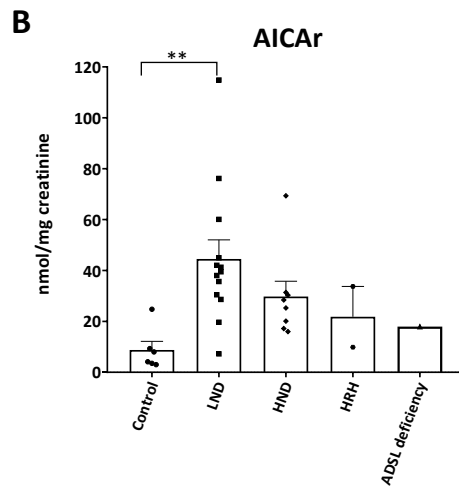

Supplement: Supplementary file 3 — Additional file 3: Figure S3. AICA and AICAr quantification in urines. PCA metabolites extraction was performed in urines from control individuals (N = 6), patients with LND (N = 14), HND (N = 8), HRH (N = 2), and ADSL deficiency (N = 1). AICA (A) and AICAr (B) levels were determined by HPLC, and the results expressed as nmol/mg creatinine. Graphs represent the mean ± SEM. *P < 0.05 **P < 0.01. One-way ANOVA, Fisher’s multiple comparison test. [file 10020_2023_774_MOESM3_ESM.pdf]

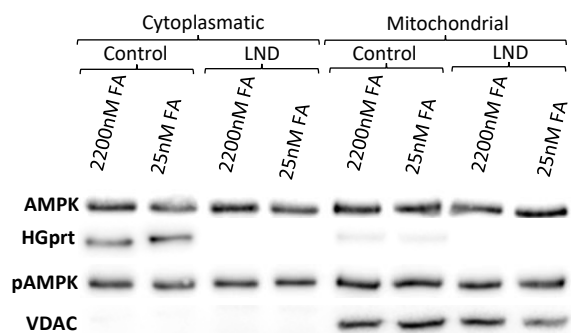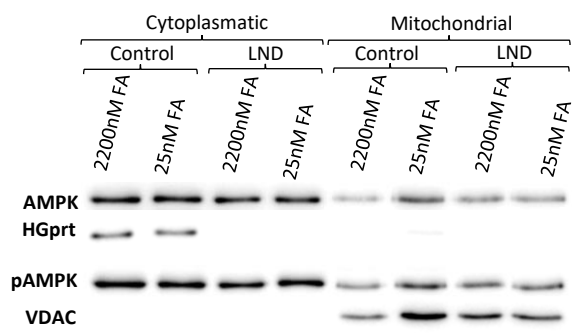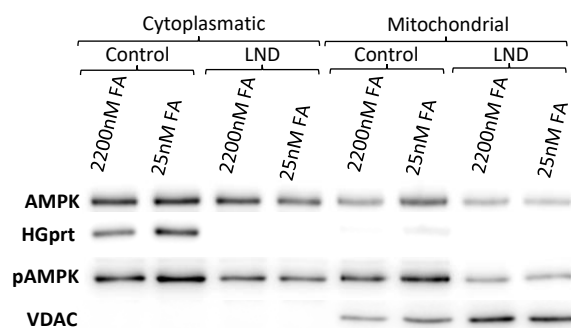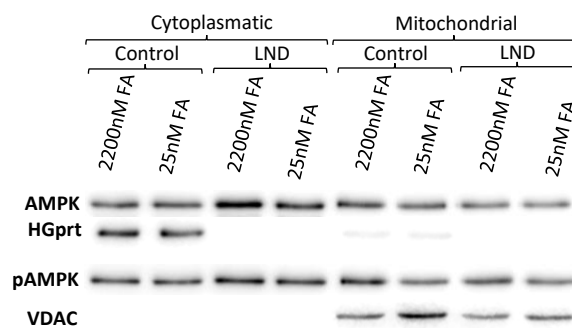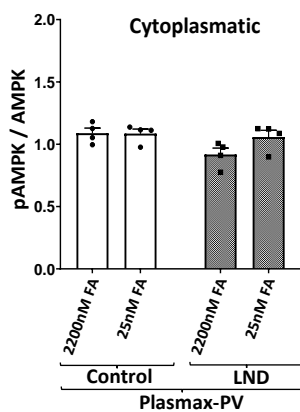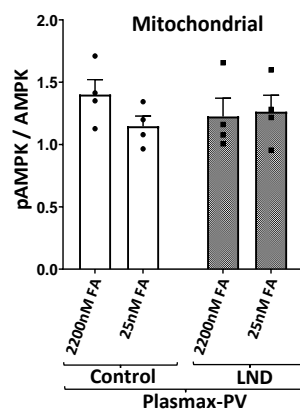

Supplement: Supplementary file 4 — Additional file 4: Figure S4. Mitochondrial and cytoplasmatic AMPK activity is similar in control and LND fibroblasts. pAMPK and AMPK levels were determined by Western blot in cytoplasmatic and mitochondrial fractions obtained from control and LND fibroblasts cultivated with Plasmax-PV containing different levels of FA. Voltage-dependent anion channel (VDAC) was used as a mitochondrial marker and the hypoxanthine–guanine phosphoribosyltransferase enzyme (HGprt) as a cytosolic marker present only in the cytoplasm of control fibroblasts but not in LND. AMPK activity expressed as the ratio pAMPK/AMPK is represented in the graph. The results are the mean ± SEM of 4 controls and 4 LND patients. 30 µg of protein were loaded per well. Two-way ANOVA, Fisher’s multiple comparison test shows no significant differences. [file 10020_2023_774_MOESM4_ESM.pdf]

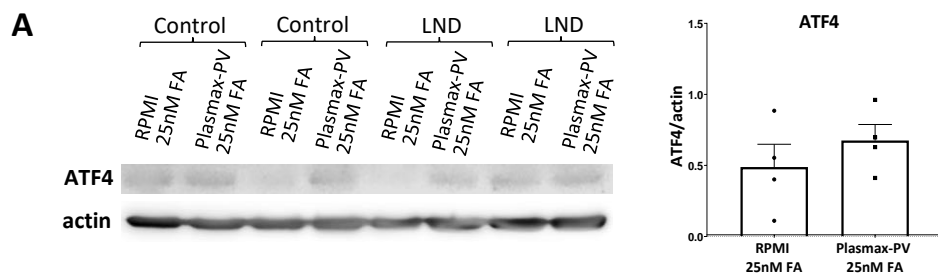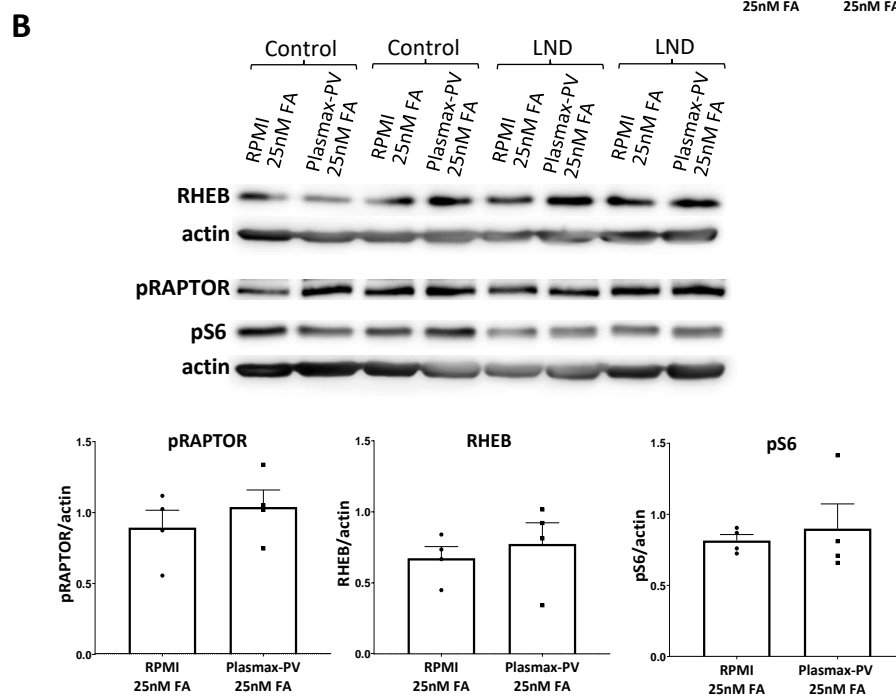

Supplement: Supplementary file 5 — Additional file 5: Figure S5. Plasmax-PV does not increase ATF4 expression and does not alter mTORC1 activity compared with RPMI. Fibroblasts were incubated for 7 days in Plasmax-PV or RPMI medium with 15% FBS containing 25 nM FA. Total cell extracts were obtained, and the expression of ATF4 (A) and the mTORC1 related proteins RHEB, pRAPTOR and pS6 (B) were determined by Western blot, quantified, and normalized by actin levels. 80 µg of protein were loaded per well. The graph represents the mean ± SEM of 4 individuals (2 controls and 2 patients with LND). Paired t-test shows no significant differences. [file 10020_2023_774_MOESM5_ESM.pdf]

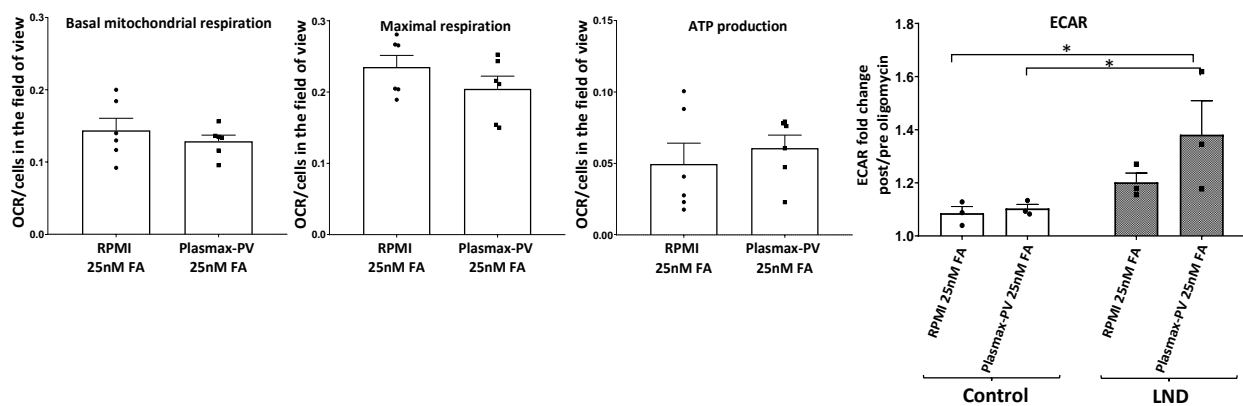

Supplement: Supplementary file 6 — Additional file 6: Figure S6. Oxygen consumption rate (OCR) is similar in fibroblasts maintained with RPMI and Plasmax, but not glycolytic capacity in LND fibroblasts. Mitochondrial respiration was evaluated in control and LND fibroblasts cultivated with RPMI or Plasmax-PV containing physiological levels (25 nM) of FA by seahorse assay as described in Material and Methods. Basal mitochondrial respiration, maximal respiration and ATP production are expressed as the oxygen consumption rate (pmol/min) normalized by the number of cells in the field of view. The results are the mean ± SEM of 6 individuals (3 controls and 3 LND). A paired t-student test shows no significant differences. Extracellular acidification rate (ECAR), expressed as the fold change after oligomycin treatment, is increased in LND fibroblasts maintained with Plasmax-PV. The results are the mean ± SEM of 3 controls and 3 LND patients. *P < 0.05. Fisher’s multiple comparison test. [file 10020_2023_774_MOESM6_ESM.pdf]
